# The associations between optimism, personal growth initiative and the latent classes of social media addiction

**DOI:** 10.3389/fpsyg.2024.1429101

**Published:** 2025-02-13

**Authors:** Heng Yue, Shiwen Gao, Yufeng Huang, Xuemin Zhang

**Affiliations:** ^1^School of Journalism and Communication, Xiamen University, Xiamen, Fujian, China; ^2^College of Physical Education, Inner Mongolia Normal University, Hohhot, Inner Mongolia, China; ^3^College of Business and Public Management, Wenzhou-Kean University, Wenzhou, China; ^4^School of Humanities and International Education, Baotou Medical College, Baotou, Inner Mongolia, China

**Keywords:** optimism, personal growth initiative, social media addiction, latent class analysis, protective effect

## Abstract

**Introduction:**

Most previous studies have explored the risk antecedents of social media addiction, while few studies have focused on the protective factors that may decrease the risk of this behavioral disorder. By using a person-centered method, the current study focused on the effects of two protective factors (optimism and personal growth initiative) on the latent classes of social media addiction.

**Methods:**

552 college students (248 females) participated in the current study, Mplus 8.3 software was adopted to conduct the latent class analysis.

**Results:**

The results revealed that based on the scores of the Bergen Social Media Addiction Scale, social media users were classified into three subgroups: low-risk class, moderate-risk class, and high-risk class. The prevalence rate of social media addiction in college students was about 9.6%. Female gender was a positive predictor of the membership of a higher-risk class. Individuals with greater optimism and personal growth initiative were less likely to be classified in a higher-risk class.

**Discussion:**

Mental health professionals or educators can use these results to design interventions targeting the two aspects to mitigate social media addiction, which may contribute to the positive development of young people.

## 1 Introduction

Social media refers to Internet-based platforms or applications that can be used for producing, sharing, and collaborating on content online (Yue et al., 2023). Since it includes various programs (such as Facebook, Twitter, Wechat, QQ, and TikTok) that can provide us with more opportunities to interact with others, share information, and find entertainment, nowadays, social media has become an indispensable part of our daily lives, and some people even regard this useful tool as a way of being and relating (Kuss and Griffiths, 2017). Although social media use makes our lives rich and colorful, the negative effects of its prevalence have raised concerns among researchers and the public. One of the hot topics that has been most extensively discussed and studied is social media addiction.

Social media addiction is characterized by being excessively concerned about social media, highly motivated, and devoting an enormous amount of time and effort to utilize social media, to the extent that an individual’s social connections, work, studies, and/or physical and mental health are impaired (Sun and Zhang, 2021). Because previous studies have convincingly revealed that social media addiction can bring about various negative consequences, many scholars have explored the risk antecedents of this behavioral disorder. However, there are few studies focused on the protective factors that may decrease the risk of social media addiction. Since protective factors can not only help individuals foster abilities to resist behavioral disorders but also facilitate their successful developments by mitigating the impact of unfavorable risk factors (Piko and Kovács, 2010). Therefore, exploring the effects of protective factors on social media addiction may not only provide more information for the related research concerning these variables but also contribute to the prevention and interference of this addictive behavior. The literature review indicates that optimism and personal growth initiative are two potential favorable factors.

Optimism is described as a stable predisposition that people anticipate positive rather than adverse events will happen to them (Genecov and Seligman, 2023). Previous studies have shown that, when facing adversity or difficulty, optimistic individuals may engage in active coping rather than avoidance and take measures to secure their health instead of indulging in health-damaging behaviors (Carver et al., 2010). Optimism has been proven to be negatively related to psychological distress such as depression, loneliness, rumination, and other negative experiences (Rincón Uribe et al., 2022). According to the assumptions of the compensatory internet use theory (Kardefelt-Winther, 2014), the lower the negative emotions people have, the less likely they will be addicted to social media. Besides, some scholars have also indicated that people who are high in optimism tend to have higher levels of social support (Liu et al., 2022) and psychological resilience (Miranda and Cruz, 2022); when facing negative life events, they often tend to use positive coping styles (Agbaria and Abu Mokh, 2022). These factors have been confirmed to be helpful for decreasing the likelihood of being addicted to social media (Cao et al., 2022; Qi et al., 2024). In addition, previous studies have revealed that optimistic individuals often have positive thoughts about themselves and the world, they often use adaptive strategies to cope with the difficult circumstances, this may buffer against the impacts of stressful events and protect themselves from pathological symptoms and maladaptive behaviors (Ning et al., 2021; Rincón Uribe et al., 2022). As maintained by the cognitive-behavioral model of social media addiction, optimistic people are less likely to use social media to escape from real life, and this will decrease the likelihood of becoming addicted to social media (Ahmed and Vaghefi, 2021). Since empirical studies have confirmed that optimism is negatively associated with social media and smartphone addiction (González-Nuevo et al., 2022; Guan et al., 2023). The current study considers that optimism may be negatively correlated with social media addiction.

Personal growth initiative refers to the active and intentional engagement in the process of personal growth, and this personal growth may be cognitive, behavioral, or affective and may happen in every life domain (Robitschek, 2003). Personal growth initiative comprises four primary abilities that are necessary for individual’s positive development: readiness for change, planfulness, using resources, and intentional behavior (Robitschek et al., 2012; Robitschek et al., 2022). Previous studies have indicated that personal growth initiative is conducive to buffering the negative impacts of adversities and relieving depression (Li et al., 2024a), seeking proper solutions to the circumstances faced and improving the adaptation abilities to different conditions (Fan et al., 2024; Liu et al., 2024; Weigold et al., 2024). In such a manner, personal growth initiative may serve as a protective factor against social media addiction. Besides, some scholars have confirmed that personal growth initiative can contribute to the satisfaction of individuals’ cognition and affect needs (Gong et al., 2024). According to the use and gratification theory and the self-determination theory (Deci and Ryan, 2000; Katz et al., 1973), the fulfillment of basic psychological needs may decrease the likelihood of being social media addicts. In addition, optimism and personal growth initiative can effectively reduce psychopathological disorders such as depression, social anxiety, traumatic stress symptoms, and so on (Li et al., 2024a; Mo et al., 2022), and enhance positive psychological experiences such as life satisfaction, self-efficacy, self-esteem, and positive affect (Weigold et al., 2020). In light of the compensatory internet use theory (Kardefelt-Winther, 2014), this may also protect users from bad usage habits. Since prior scholars have confirmed that personal growth initiative is a negative predictor of addictive behaviors such as drinking behavior, internet gaming disorder, and smartphone addiction (Chen and Guo, 2022; Gan et al., 2022; Tao et al., 2024), in the current study, it is possible that personal growth initiative can significantly and negatively predict social media addiction.

In terms of the research method, most of the prior studies have been conducted based on variable-centered methods. Although variable-centered studies provide valuable information, because they do not consider individual characteristics, these studies have been questioned for obscuring diversity and fostering inaccurate conclusions (Von Eye and Bergman, 2003). Under this circumstance, researchers turn to person-centered methods. Since this type of statistical technique does not presume that personal characteristics are the same in all people, researchers can obtain a greater knowledge of personal characteristics among the study population (Kongsted and Nielsen, 2017). One such statistical method is Latent Class Analysis (LCA). LCA is a useful statistical technique that can identify the “hidden groups” of people who exhibit similar score patterns on the observed indicators, while the score patterns of different subgroups differ as much as possible (Nylund-Gibson and Choi, 2018). Since LCA provides some evaluative indicators that can be used to determine the optimal number of latent classes, this method is considered more precise and objective but less arbitrary or subjective than other cluster analysis techniques (Schreiber, 2017). Some scholars also use LCA to identify the cut-off points for measurement instruments, such as the 10-item center for epidemiologic studies depression scale (Fu et al., 2022) and the 8-item online social networking addiction scale (Li et al., 2020). The cut-off values can be used for calculating the prevalence rate of behavioral or psychopathological disorders as well. In current scientific research, this method has been widely used by researchers in various areas such as psychology (Luo et al., 2021), psychiatry (Dahmer et al., 2022), and even engineering disciplines (Li et al., 2019).

Although prior studies have used LCA to identify subgroups of social media addiction, their results are not consistent with each other (Bányai et al., 2017; Li et al., 2020; Luo et al., 2021; Peng and Liao, 2023). For this reason, further exploring the latent classes of this behavioral disorder may provide researchers with more knowledge for identifying the addictive usage patterns and the prevalence rate precisely. Besides, since the benefits of protective factors have been elucidated by one previous study (Piko and Kovács, 2010), and the evidence provided by the literature indicates that optimism and personal growth initiative may be the protective factors of this addictive behavior. Therefore, the current study aimed to investigate the latent classes of social media addiction and the protective effects of optimism and personal growth initiative.

## 2 Method

### 2.1 Participants

An online survey was conducted to collect data. The survey was administered in a classroom setting during the class intervals or the after-class time. After acquiring the permissions and informed consents from the teachers and the students, the link of the questionnaire was shared in the students’ WeChat or QQ groups. They could fill in the questionnaires with no restriction. The criteria for excluding the invalid questionnaires were: (1) the answering time less than 75 s; (2) selecting the same answer for all the items; (3) choose a wrong option in the attention check items.

In total, 622 Chinese college students in Fujian and Zhejiang province and Inner Mongolia Autonomous Region participated in the current study. After removing 70 invalid cases, the final sample included 552 undergraduate and graduate students. There were 304 males and 248 females. The average age of the participants was 20.52 years (SD = 1.77), ranging from 18 to 26 years.

### 2.2 Measurements

#### 2.2.1 Optimism

The Life Orientation Test (Wen, 2012) was administered as a self-reported measure of optimism. This instrument had six items; all the items were measured on a five-point Likert scale ranging from 0 (strongly disagree) to 4 (strongly agree). A higher total score indicated a higher level of optimism. In the current study, the Cronbach’s *α* of this scale was 0.860.

#### 2.2.2 Personal growth initiative

Personal Growth Initiative was measured using the Personal Growth Initiative Scale–II (Robitschek et al., 2012). This instrument had 16 items; all the items were measured on a six-point Likert scale ranging from 0 (strongly disagree) to 5 (strongly agree). A higher total score indicated a higher level of personal growth initiative. In the current study, the Cronbach’s α of this scale was 0.904.

#### 2.2.3 Social media addiction

Social media addiction was evaluated by the Bergen Social Media Addiction Scale (BSMAS) (Luo et al., 2021). This scale consisted of 6 items; each item was rated using a five-point Likert scale, ranging from 1 (very rarely) to 5 (very often). Higher total scores indicated greater levels of social media addiction. In this study, the Cronbach’s α of this scale was 0.854.

### 2.3 Statistical analysis

SPSS 25.0 was used for descriptive statistics, correlation analysis, and logistic regression analysis. Mplus 8.3 was employed to conduct the LCA. Some indices were employed to determine the optimal number of latent classes: the akaike information criterion (AIC), the bayesian information criterion (BIC), the sample size-adjusted BIC (aBIC), the Entropy, the vuong-lo–mendell–rubin likelihood ratio (LRM) test, and the bootstrapped likelihood ratio tests (BLRT). For the first three indices, lower values indicated a better fit for the model. Entropy indicated how accurately the model defined classes; an entropy value greater than 0.8 was acceptable, and a value close to 1 was ideal. Both the LMR and BLRT provided *p* values; a significant *p* value indicated that the current model (*k* classes) was statistically better than the former model (*k*-1 classes) (Weller et al., 2020).

## 3 Results

### 3.1 Descriptive statistics

The results of the descriptive statistics for the study variables were displayed in Table 1. As seen, optimism (*r* = −0.348, *p* < 0.01) and personal growth initiative (*r* = −0.305, *p* < 0.01) were negatively correlated with social media addiction, suggesting that individuals who had higher levels of positive psychological characteristics were less likely to experience social media addiction. The positive relationship between optimism and personal growth initiative was significant (*r* = 0.457, *p* < 0.01), indicating that optimism and personal growth initiative might reinforce each other.

**Table 1:** The results of descriptive statistics of the study variables.

Variables	Mean ± SD	OPT	PGI	SMA
OPT	18.02 ± 5.26	1		
PGI	60.26 ± 15.56	0.457**	1	
SMA	15.38 ± 4.97	−0.348**	−0.305**	1

OPT, optimism; PGI, personal growth initiative; SMA, social media addiction. **p < 0.01.

### 3.2 Primary LCA results

The fit indices yielded by the LCA were presented in Table 2. The AIC and aBIC values declined for all models up to the fifth-class solution. The BIC value of the 3-class was slightly larger than the 4-class model, but both the two BIC values were smaller than the values of other models, implying that the two models could be used as the optimal solution. The entropy values for the 2-class and 3-class models were higher than 0.80, but the entropy values for the 4-class and 5-class models were smaller than this cutoff value. Indicating that the 4-class and 5-class models might be unacceptable. The *p* values yielded by LMR and BLRT could be used to select a final model. Although all the *p* values provided by the BLRT were smaller than 0.01, the results demonstrated that the *p* value provided by the LMR test was not significant after 3 classes, indicating that the 4-class solution was not significantly better than the 3-class solution. Therefore, the 3-class LCA model was considered the best-fitting model.

**Table 2:** Latent class analysis model comparisons.

Latent class	K	AIC	BIC	aBIC	Entropy	LMR	BLRT
1	12	9975.02	10026.78	9988.69	—	—	—
2	19	9195.77	9277.73	9217.41	0.832	<0.01	<0.01
3	26	8714.73	8826.89	8744.35	0.868	<0.01	<0.01
4	33	8682.24	8824.58	8719.83	0.745	0.08	<0.01
5	40	8658.82	8831.36	8704.38	0.783	0.45	<0.01

**Figure 1:**
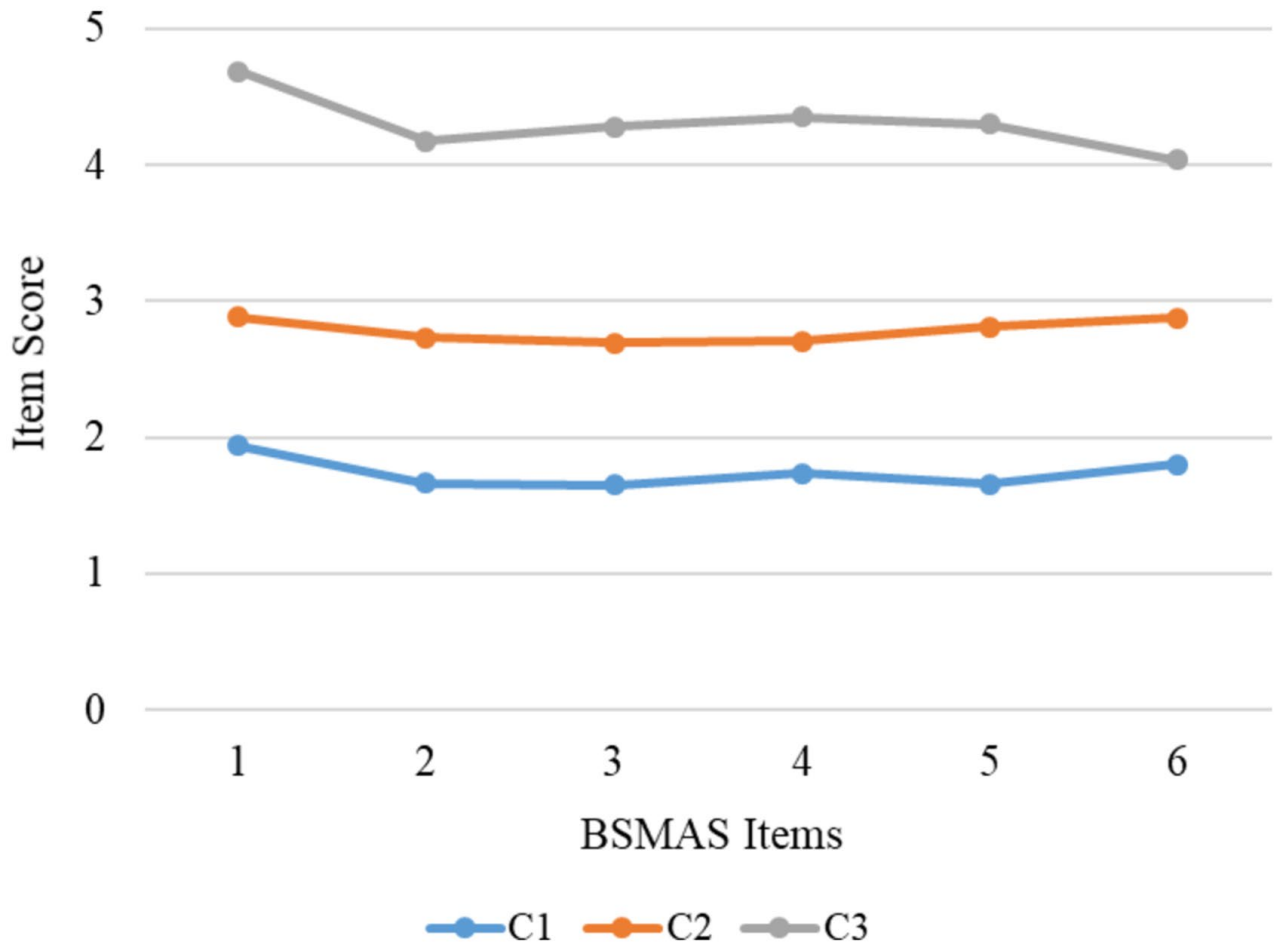
The three classes identified from the latent class analysis.

Figure 1 presented the latent classes based on the item scores. Members of Class 1 (C1, 34.96% of the entire sample, 193 individuals) exhibited a low risk of social media addiction, as demonstrated by the lowest scores on all the items. This class was named “low-risk class.” Members of Class 2 (C2, 55.44% of the entire sample, 306 individuals) had higher scores than those of Class 1, while they had lower scores than those of Class 3. This class was named “moderate-risk class.” Members of Class 3 (C3, 9.6% of the entire sample, 53 individuals) had the highest scores on all the items; therefore, this class was named “high risk class.”

### 3.3 Comparisons of the study variables across latent classes

The outcomes of the comparisons of the study variables across the three latent classes were presented in Table 3. The *post hoc* analyses of the ANOVA demonstrated that the scores of optimism and personal growth initiative of the high-risk class were significantly lower than the other two classes. Additionally, scores of the two variables in the moderate-risk class were significantly lower than those in the low-risk class. Conversely, social media addiction score in a higher risk class was significantly greater than in a lower risk class. All these results indicated that LCA was an effective approach for distinguishing the differences between various subgroups.

**Table 3:** Comparisons of the study variables across latent classes.

Variables	Low risk class	Moderate class	High risk class	*F*	*Post hoc*
OPT	19.84 ± 4.65	17.43 ± 5.32	14.81 ± 4.80	25.471**	C1 > C2 > C3
PGI	64.59 ± 13.77	59.53 ± 15.86	49.72 ± 14.19	21.660**	C1 > C2 > C3
SMA	10.31 ± 1.96	16.76 ± 2.02	25.89 ± 2.00	1,428.216**	C3 > C2 > C1

OPT, optimism; PGI, personal growth initiative; SMA, social media addiction. C1, Class1. C2, Class2. C3, Class 3. **p < 0.01.

### 3.4 Effects of study variables on latent classes

A multinomial logistic regression analysis was performed to explore the effects of optimism and personal growth initiative on the latent classes of social media addiction. The results were displayed in Table 4. Compared with the low-risk class, age had no significant effects on the likelihood of being in a particular cluster; while gender (female) had a marginally significant effect on predicting the membership of the moderate-risk class (*OR* = 1.407, *p* = 0.080) and a significant effect on predicting the membership of the high-risk class (*OR* = 2.485, *p* = 0.080). This indicated that compared with males, females had 40.70% marginally significantly increased possibility of being divided into the moderate-risk class; and they had 145.80% significantly increased possibility of being classified into the high-risk class.

**Table 4:** Results of multinomial logistic regression (using low-risk class as the reference class).

	Moderate risk class					High risk class				
Variables	*B*	SE	Wald	Exp (B)	*p*	*B*	SE	Wald	Exp (B)	*p*
Age	−0.022	0.055	0.164	0.978	0.685	0.014	0.089	0.025	1.014	0.875
Gender	0.341	0.195	3.073	1.407	0.080	0.910	0.339	7.210	2.485	0.007
OPT	−0.081	0.021	15.211	0.922	<0.01	−0.144	0.037	15.305	0.866	<0.01
PGI	−0.013	0.007	3.260	0.987	0.071	−0.046	0.012	14.025	0.955	<0.01

OPT, optimism; PGI, personal growth initiative; SMA, social media addiction. C1, Class1. C2, Class2. C3, Class 3.

Personal growth initiative had marginally significant impacts on predicting the membership of the moderate-risk class (*OR* = 0.987, *p* = 0.071), while it could significantly predict the membership of the high-risk class (*OR* = 0.955, *p* < 0.01). This implied that each adding point for personal growth initiative was marginally significantly related to 1.3% decreased risk of being classified into the moderate-risk class, and significantly related to 4.5% decreased likelihood of being categorized into the high-risk class.

Optimism was a valid predictor of being classified into both the moderate-risk (*OR* = 0.922, *p* < 0.01) and the high-risk classes (*OR* = 0.866, *p* < 0.01). This suggested that each adding point for optimism was associated with 7.8% decreased risk of being classified into the moderate-risk class and significantly related to 13.4% decreased likelihood of being categorized into the high-risk class.

## 4 Discussion

By employing a person-centered method (LCA), the current study examined the latent classes of social media addiction and the effects of optimism and personal growth initiative on predicting the membership of these latent clusters. The main findings and implications were presented as follows:

Based on the scores of the BSMAS, three heterogeneous clusters were identified: the low-risk class, the moderate-risk class, and the high-risk class. This result was consistent with previous studies examining the latent class of social media addiction based on different measurement instruments and various cultures (Cheng et al., 2022; Pi et al., 2024; Smith and Short, 2022). However, by using the same instrument and analysis method as the current study, some scholars have found that social media addiction can be divided into five subgroups (Luo et al., 2021; Peng and Liao, 2023). This may be because most of the participants in the present study and in studies with the same results are adults (average age is older than 20 years old), while the majority of participants in studies with heterogeneity results are adolescents (average age is about 15 years old). In fact, adolescents are often middle school or high school students; their social media use is under the supervision of their parents and teachers; however, in some technical schools, adolescents’ social media use is not restricted. Accordingly, compared with college students whose social media use is not limited, adolescents in different schools may have more complex usage models. Besides, on average, adolescents’ social media usage period is shorter than that of adults; compared with their adult counterparts, adolescents are in exploratory usage stages and may have diverse usage patterns (Martínez-Ferrer et al., 2018). Moreover, adolescence is a developmental period during which individuals’ bodies and minds may have different levels of maturation; this will also diversify their behavioral patterns and make them show more latent social media addiction clusters.

In previous studies, the latent class that has the highest risk of behavioral or psychopathological disorder was often considered the “gold standard” for calculating the prevalence rate of psychological and behavioral disorders (Bányai et al., 2017; Li et al., 2020; Luo et al., 2021). The results of the current study demonstrated that the prevalence of social media addiction among college students was about 9.6%. This value was close to the prevalence rates yielded by some previous studies that employed BSMAS and LCA as measurement instruments and analysis approaches (8 to 10%) (Cheng et al., 2021; Peng and Liao, 2023; Yue, 2023), indicating the good credibility and accuracy of this research method. However, the result was a little different from one prior study that showed the prevalence rate in a Hungarian sample (4.5%) (Bányai et al., 2017). The reason might lie in the cultural discrepancy. In individualistic cultures, independence is more emphasized, which often leads to fractured and temporary relationships between people, while in a collectivistic culture, people often place a high value on group identity and have a strong inclination to form lifelong ties (Kim et al., 2011). Therefore, compared with individualist countries, people in collectivist countries are more likely to use social media to maintain relationships, seek social support, and form consensus (Chan and Cheng, 2016; Cheng et al., 2021; Yue, 2023). All these motivations may increase their proneness to social media addiction and result in the high prevalence of this addictive behavior in collectivist countries.

The results of the ANOVA indicated that different subgroups had different levels of personal characteristics. The optimism and personal growth initiative scores of the high-risk class were significantly lower than those of the moderate- and low-risk classes. These outcomes were in line with empirical studies that found levels of optimism and personal growth initiative were negatively associated with the severity of internet addiction and smartphone addiction, respectively (Chen and Guo, 2022; Guo et al., 2020). The correlation analysis of the present study also revealed negative relationships between optimism, personal growth initiative, and social media addiction. The reason might lie in the fact that the patterns of social media addiction were similar across these clusters; thus, the score difference became the major distinction. All the evidence confirmed the validity of the LCA results. Besides, since the high-risk group had the lowest scores of optimism and personal growth initiative, this implied that improving the levels of the two factors might be effective approaches for reducing social media addiction.

The results of the logistic regression analysis suggested that, compared with the low-risk class, the female gender had significantly higher odds of being in the high-risk class and a marginally higher possibility of being in the moderate-risk class. These results corroborated findings from previous studies that showed women were more likely to be addicted to social media (Çimke and Yıldırım Gürkan, 2023). Prior researchers have found that, compared with males, females often attach more value to interpersonal relationships, and they are more likely to employ social media as an interaction instrument (Zhao et al., 2022). When social requirements are not fully fulfilled in real life, females are more likely to use social media to keep informed about others’ updates, seek attention and validation from others (Li and Zhuo, 2023). In the long run, these usage habits may contribute to social media addiction and increase the possibility of being divided into a higher-risk class. Besides, previous studies have revealed that females are more vulnerable to affective disorders such as depression, anxiety disorders and posttraumatic stress disorder (Rubinow and Schmidt, 2019). According to the preference for online social interaction theory, people with adverse feelings such as loneliness and depression prefer online social interaction; this usage habit may contribute to social media addiction (Caplan, 2003). Therefore, the more negative emotions people experience, the higher levels of social media addiction they may have; likewise, the greater likelihood they may have to be classified into the higher-risk subgroups.

The current study found that optimism was a significant and negative predictor of being in a higher-risk class. This result was consistent with previous literature showing that optimism can decrease the risk of being addicted to social media and smartphones (González-Nuevo et al., 2022; Guan et al., 2023). The reason might lie in the following aspects. Firstly, optimism has been proven to be conducive to decreasing depression, loneliness, rumination, and other negative experiences (Rincón Uribe et al., 2022). And when facing negative life events, optimists may display less psychological distress (Kagan et al., 2024). According to the compensatory internet use theory (Kardefelt-Winther, 2014), less severe negative emotions can be compensated by a few hours of social media usage, and this is accompanied by a few problematic consequences. Therefore, by alleviating negative emotions, optimistic individuals are less likely to be divided into higher-risk classes. Secondly, optimism can enhance self-control ability (Oriol et al., 2020), and as a positive emotion, optimism can also counteract the ego-depletion effect (Ma and Mo, 2022; Yvo et al., 2014). In this way, it protects people from social media addiction. Thirdly, researchers have found that optimists tend to work harder at interpersonal relationships, they are easier to be liked and often gain more social support from their surroundings (Liu et al., 2022). According to the interpersonal model of addiction relapse (Leach and Kranzler, 2013), a good interpersonal relationship is helpful for the recovery from social media addiction. Therefore, optimistic people, benefiting from high-quality interpersonal relationships, may be more likely to be divided into lower-risk classes. Bandura’s triadic reciprocal determinism model also posits that behaviors are determined by personal factors and environmental influences. In the current study, optimism and its positive consequences (such as interpersonal networks and social support) could be regarded as personal and environmental factors, respectively. And they could effectively decrease the severity of social media addiction and the likelihoods of being in the higher-risk class.

The results also indicated that, using the low-risk class as the reference class, college students with higher personal growth initiative were significantly more likely to be classified into the low-risk subgroups, and they had marginally fewer chances to be in the moderate-risk class. These findings supported the outcomes of prior studies indicating that personal growth initiative is associated with various problematic behaviors, such as internet gaming disorder (Gan et al., 2022), smartphone addiction (Chen and Guo, 2022), and so on. Possible explanations were listed as follows. Firstly, prior scholars considered that a low or moderate level of psychological distress can be effectively compensated through social media usage (Kardefelt-Winther, 2014; Yue et al., 2021), and some studies have confirmed that personal growth initiative can significantly mitigate various psychological distress such as posttraumatic stress, maladaptive rumination, social anxiety, and depression (Shigemoto et al., 2017; Weigold et al., 2020). Therefore, by relieving negative emotions, personal growth initiative can significantly decrease social media addiction and reduce the odds of being classified into higher-risk classes. Secondly, personal growth initiative has been regarded as a useful intrinsic personal resource that can promote positive development in people (Huang et al., 2023). According to the protective factor model of resilience, these internal resources (coping skills and self-efficacy) can be helpful for people to avoid, buffer, or reduce the detrimental consequences (i.e., substance use, violent behavior) of negative life experiences (Fergus and Zimmerman, 2005). Therefore, personal growth initiative can decrease the level of social media addiction. Thirdly, the interaction of person-affect-cognition-execution (I-PACE) model assumes that negative personality traits are risk factors for social media addiction (Brand et al., 2016). Personal growth initiative has been found to be significantly associated with self-esteem, self-efficacy, self-control, perceived social support, and other positive personal and social cognitive characteristics (Cai and Lian, 2022; Chen and Guo, 2022; Freitas et al., 2016). Accordingly, by improving positive personal characteristics, personal growth initiative effectively reduces social media addiction and lower the odds of being distributed to higher-risk classes.

## 5 Implications

Firstly, this study indicates that optimism and personal growth initiative are protective factors of social media addiction. Mental health professionals or educators can use these results to design interventions targeting the two aspects to mitigate social media addiction, which may contribute to the positive development of young people. Secondly, instead of concentrating on the risk factors, this study focuses on the protective factors of social media addiction, which may provide some useful ideas and directions for future studies. Thirdly, the prevalence of social media addiction yielded by the current study and some previous studies indicate that the results of LCA have good stability, credibility, and accuracy, especially in the same cultural context. Therefore, researchers and educators can use this method to preliminary identify the “positive individuals” of social media addiction, which may contribute to the prevention and timely intervention of this behavioral disorder.

## 6 Limitations

This study has some limitations. Firstly, since optimism has been studied in the context of anxiety, depression (Yıldırım and Çiçek, 2022), post-traumatic stress disorder (Li et al., 2024b), and suicide attempts (Snooks and McLaren, 2022), it is necessary to consider the psychiatric variables linked to optimism as potential confounding variables. This may provide more robust evidence for understanding the associations between the study variables. Secondly, likewise, optimism has been associated with physical health (Rasmussen et al., 2009); it can be hypothesized that optimism may decrease and social media addiction may increase in physically immobile individuals. Therefore, variables related to physical health are also needed to be taken into consideration, which may be helpful for clarifying the associations between the study variables and providing research directions for future studies in this area. Thirdly, since personal growth initiative has been studied in patients with anxiety and depression (Weigold et al., 2020), the two psychological disorders are frequently comorbid with social media addiction; thus, cases with clinical diagnoses should be excluded from the study. Fourthly, there are some limitations in the sample’s characteristics, such as the age of the sample only ranged from 18 to 26 years old, this study is conducted in a Chinese context, and all the participants are college students and so on. These limitations may restrict the generalization of the results. Future studies are encouraged to use samples with wider age ranges, educational and cultural backgrounds, which may provide more empirical evidence for the application and generalization of the outcomes. At last, because the current study uses a cross-sectional design, this may limit causal inference. Accordingly, future studies are encouraged to use longitudinal design to provide more useful information concerning the associations between these study variables.

## 7 Conclusion

Based on the scores of BSMAS and by using LCA, the current study examined the latent classes of social media addiction and two protective factors: optimism and personal growth initiative. The results demonstrated that: (1) social media users could be classified into three latent classes: the low-risk class, the moderate-risk class, and the high-risk class. Individuals’ positive personal characteristics tend to be higher in a lower-risk subgroup; (2) the prevalence rate of social media addiction among college students was about 9.6%; (3) female gender was a positive predictor of membership in a higher-risk class; and (4) individuals with greater optimism and personal growth initiative were less likely to be classified in a higher-risk class.

## Data Availability

Data presented in this study are available from the corresponding author on reasonable request and with the permission of the Research Ethics Committee.
